# Exploring the consequences of food insecurity and harnessing the power of peer navigation and mHealth to reduce food insecurity and cardiometabolic comorbidities among persons with HIV: protocol for development and implementation trial of *weCare/Secure*

**DOI:** 10.1186/s13063-022-06924-3

**Published:** 2022-12-12

**Authors:** Amanda E. Tanner, Deepak Palakshappa, Caryn G. Morse, Lilli Mann-Jackson, Jorge Alonzo, Manuel Garcia, Elena Wright, Ajay Dharod, Scott Isom, Ana D. Sucaldito, Lucero Refugio Aviles, Scott D. Rhodes

**Affiliations:** 1 Department of Public Health Education, University of North Carolina Greensboro, Coleman 437E, Greensboro, NC 27402 USA; 2 Department of Internal Medicine, Wake Forest University School of Medicine, Winston-Salem, USA; 3 Department of Pediatrics, Wake Forest University School of Medicine, Winston-Salem, USA; 4 Department of Epidemiology and Prevention, Wake Forest University School of Medicine, Winston-Salem, USA; 5 Section on Infectious Diseases, Department of Internal Medicine, Wake Forest University School of Medicine, Winston-Salem, USA; 6 Department of Social Sciences and Health Policy, Wake Forest University School of Medicine, Winston-Salem, USA; 7 Department of Implementation Science, Wake Forest University School of Medicine, Winston-Salem, USA; 8 Department of Internal Medicine, Informatics and Analytics, Wake Forest University School of Medicine, Winston-Salem, USA; 9 Wake Forest Center for Healthcare Innovation, Wake Forest University School of Medicine, Winston-Salem, USA; 10 Wake Forest Center for Biomedical Informatics, Wake Forest University School of Medicine, Winston-Salem, USA; 11 Department of Biostatistics and Data Science, Wake Forest University School of Medicine, Winston-Salem, USA

**Keywords:** HIV, Food insecurity, Diabetes, Peer navigation, mHealth, Feasibility studies, United States

## Abstract

**Background:**

Food insecurity, or the lack of consistent access to nutritionally adequate and safe foods, effects up to 50% of people living with HIV (PWH) in the United States (US). PWH who are food insecure have lower antiretroviral adherence, are less likely to achieve viral suppression, and are at increased risk developing of serious illnesses, including cardiometabolic comorbidities. The objectives of this study are to better understand how food insecurity contributes to the development of cardiometabolic comorbidities among PWH and to test a novel bilingual peer navigation-mHealth intervention (weCare/Secure) designed to reduce these comorbidities in food-insecure PWH with prediabetes or Type 2 diabetes (T2DM).

**Methods:**

In Aim 1, we will recruit a longitudinal cohort of 1800 adult (≥18 years) PWH from our clinic-based population to determine the difference in the prevalence and incidence of cardiometabolic comorbidities between food-secure and food-insecure PWH. Food insecurity screening, indicators of cardiometabolic comorbidities, and other characteristics documented in the electronic health record (EHR) will be collected annually for up to 3 years from this cohort. In Aim 2, we will conduct a randomized controlled trial among a sample of food-insecure PWH who have prediabetes or T2DM to compare changes in insulin sensitivity over 6 months between participants in weCare/Secure and participants receiving usual care. In Aim 3, we will conduct semi-structured individual in-depth interviews to explore the effect of the intervention among intervention participants with varying insulin sensitivity outcomes.

**Trial status:**

Aim 1 (longitudinal cohort) recruitment began in May 2022 and is ongoing. Aim 2 (intervention) recruitment is planned for spring 2023 and is expected to be completed in spring 2024. Aim 3 (process evaluation) data collection will occur after sufficient completion of the 6-month assessment in Aim 2. Final results are anticipated in fall 2025.

**Conclusions:**

This research seeks to advance our understanding of how food insecurity impacts the development of cardiometabolic comorbidities among PWH and how food insecurity interventions may alleviate relevant comorbidities. Given the growing interest among health systems in addressing food insecurity, if the intervention is found to be efficacious, it could be broadly disseminated across HIV clinical care settings.

**Trial registration:**

ClinicalTrials.gov NCT04943861. Registered on June 29, 2021.

The social determinants of health (SDH), or the circumstances in which people are born, work, live, and age, have a profound impact on morbidity and mortality [1–5]. SDH can negatively affect health by leading to adverse social risk factors such as food insecurity, or the lack of consistent access to nutritionally adequate and safe foods. In 2021, 10.2% of United States (US) households, or over 30 million people, were food insecure, and nearly 90% of the US counties with the highest rates of food insecurity were in the South [6], particularly affecting people living in rural communities [7–9]. Compared to food-secure individuals, food-insecure individuals are more likely to have worse diet quality, higher smoking rates, and less physical activity, and food insecurity is associated with type 2 diabetes mellitus (T2DM), hypertension, and cardiovascular disease [10–14]. Existing research describes factors influencing food insecurity and cardiometabolic comorbidities through the pathways outlined in Fig. 1: nutritional, mental health, and behavioral [15, 16].

**Fig. 1:**
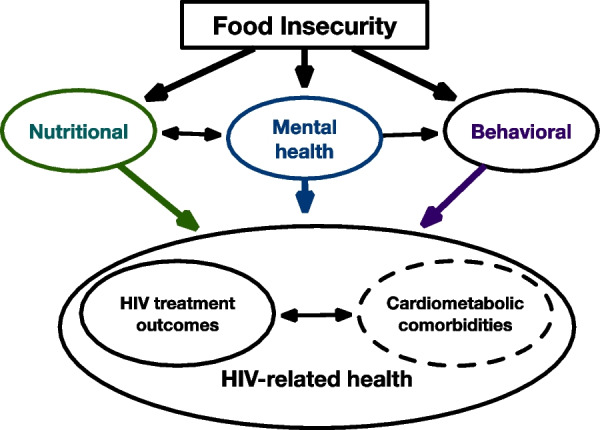
Food insecurity co-morbidity conceptual model

People living with HIV (PWH) are particularly at risk for the detrimental effects of food insecurity; up to 50% of PWH in the US are estimated to be food insecure [11–13]. PWH who are food insecure have lower antiretroviral adherence, are less likely to achieve viral suppression, and have an increased incidence of serious illnesses and mortality [12, 17–19].

Although food insecurity is associated with cardiometabolic comorbidities in people without HIV, little is known about how food insecurity affects the development of these comorbidities among PWH [20–22]. At the same time, there is a critical need for interventions to reduce food insecurity among PWH. Many national healthcare organizations, including the National Academy of Medicine, recommend that clinicians and health systems identify and address food insecurity as part of routine clinical care [23–25]. Interventions are being implemented to address food insecurity in primary care settings [26–28], but there are few evidence-based interventions in clinical settings caring for PWH [13, 26–28]. An effective strategy to address food insecurity in PWH could be disseminated broadly across clinics and health systems to improve the care and health of PWH.

Peer navigation to support access to food has the potential to reduce cardiometabolic risk through reducing food insecurity among PWH. Natural helping (e.g., peer navigation, lay health advising, and peer support) is gaining attention as a strategy to improve health outcomes in underserved or vulnerable communities [29–44], and the strategy holds particular promise for reducing cardiometabolic risk among PWH. Peer navigation differs from patient or clinic-based navigation; peer navigators share a unique understanding of the population they are working with, based on shared experiences and/or sociodemographic characteristics. Additionally, mHealth is a powerful tool for peer navigators to use with PWH. Social media use, including Facebook, Instagram, texting, and GPS-based mobile applications (“apps”) designed for social and sexual networking, have been increasing in the US, due in part to the proliferation of mobile devices [45–48]. PWH are often socially and geographically isolated [49, 50], making mHealth approaches that utilize social media a useful strategy for reaching PWH and providing peer navigation. mHealth can shrink the distance between individuals needing support and those who can provide support.

Accordingly, the objectives of this study are to better understand how food insecurity contributes to the development of cardiometabolic comorbidities among PWH and to test a novel bilingual peer navigation-mHealth food insecurity intervention (weCare/Secure) designed to reduce these comorbidities among food-insecure PWH living in the southeastern United States.

## Methods

### Ethics statement

The Institutional Review Board (IRB) at the Wake Forest University School of Medicine is the IRB of record. The Wake Forest University School of Medicine IRB has reviewed and approved all the procedures outlined in this protocol. The study is registered with ClinicalTrials.gov (NCT04943861).

### Study settings

This protocol is being implemented at the Wake Forest Infectious Diseases Specialty Clinic, a clinic within an academic medical center that provides HIV medical care for over 2000 patients with multiple comorbidities and significant psychosocial complexity annually. The population consists of a mix of uninsured patients and patients with private and public insurance (e.g., Medicaid). The clinical catchment area includes a 40-county area of largely rural North Carolina, Virginia, West Virginia, and Tennessee. The clinic is comprised of PWH from particularly marginalized communities; 67% are African American/Black; 9% are Latine; 50% are 50 years old or older; 35% are uninsured; and many are low income and have limited educational attainment.

### Overall study design

**Fig. 2:**
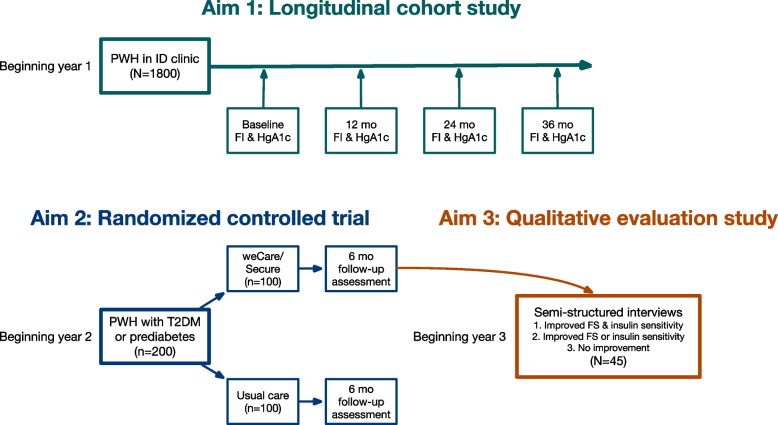
Overall study design for food insecurity and cardiometabolic comorbidities among persons living with HIV

The objective of this study is to better understand the relationship between food insecurity and cardiometabolic comorbidities and to test the effect of a food insecurity intervention (weCare/Secure) on insulin sensitivity among PWH with prediabetes or T2DM. In Aim 1, we will recruit a longitudinal cohort of 1800 from our clinic-based population of adult PWH to determine the difference in the prevalence and incidence of cardiometabolic comorbidities between food-secure and food-insecure PWH (Fig. 2).

Food insecurity screening, indicators of cardiometabolic comorbidities, and other characteristics documented in the electronic health record (EHR) will be collected annually for up to 3 years from this cohort. In Aim 2, we will conduct a randomized controlled trial (RCT) among a sample of food-insecure PWH who have prediabetes or T2DM to compare changes in insulin sensitivity over 6 months between participants in weCare/Secure and participants receiving usual care. In Aim 3, we will conduct semi-structured individual in-depth interviews to understand the effect of the intervention on insulin sensitivity among intervention participants.

#### Aim 1: Use a longitudinal cohort to determine the difference in the prevalence and incidence of cardiometabolic comorbidities between food-secure and food-insecure PWH

##### Participants

To be eligible to participate in Aim 1, a participant must (a) be a patient of the Wake Forest Infectious Diseases Specialty Clinic, (b) be living with HIV, (c) be ≥18 years of age, and (d) provide informed consent. PWH will be excluded if they are unable to speak English or Spanish or have cognitive impairment that would prevent participation. Aim 1 participants will be compensated $20 at each data collection period.

##### Data collection

For this aim, we will recruit, enroll, and collect data from a longitudinal cohort of PWH (*N* = 1800); data will be collected at baseline and at 12-, 24-, and 36-month follow-up. We will implement the Mobile Patient Technology for Health (mPATH™) in the clinic. mPATH™ is a patient-facing digital health platform allowing patients to confidentially answer routine ambulatory screening questions [51]. At the time of ambulatory visit check-in, patients are provided a tablet by front desk staff with mPATH™ that includes standard clinic assessments of fall risk, depression, and anxiety, and incorporates the 2-item Hunger Vital Sign measure [52, 53], which measures household food insecurity over the prior 12 months. Because people at risk of food insecurity often have other unmet social needs, mPATH™ also assesses housing stability and transportation barriers (Table 1) [54]. The mPATH™ platform offers data collection in English or Spanish. If a participant is unable to effectively utilize mPATH™, a clinic staff member will assist by reading the items to the participant. Data from mPATH™ flows directly into the participant’s EHR.

**Table 1:** Study measures

Domain	Measures and citations	Data source
*Aim 1 and Aim 2*		
Prediabetes or T2DM (primary outcome)	Fasting and non-fasting glucose, A1C level, diagnosis codes (ICD-10), current diabetic medications (including glucagon-like peptide 1 agonist, sodium-glucose cotransporter 2 inhibitor, dipeptidyl peptidase 4 inhibitor, sulfonylurea, incretin mimetic, or insulin)	EHR data extraction (baseline, 12mo, 24mo, 36mo)
Hypertension, hyperlipidemia, obesity, and elevated waist circumference (secondary outcomes)	History of high blood pressure, current blood pressure-lowering medications, 3 most recent systolic and diastolic blood pressure readings, history of high cholesterol, current cholesterol-lowering medications, lipid panel (total, LDL, HDL, triglycerides), body-mass index, waist circumference	EHR data extraction (baseline, 12mo, 24mo, 36mo)
Food insecurity	2-item Hunger Vital Sign [50, 51]	EHR data extraction (baseline, 12mo, 24mo, 36mo)
Basic demographics	Age, race, ethnicity, gender, sexual orientation, insurance status, preferred language, zip codes	EHR data extraction (baseline, 12mo, 24mo, 36mo)
Health status	Time since HIV diagnosis and since ART initiation, current HIV medications (including ART), other relevant medications (e.g., oral corticosteroids), pregnancy status, smoking status, other medical histories	EHR data extraction (baseline, 12mo, 24mo, 36mo)
Other unmet social needs	Housing stability, transportation barriers to obtaining medical care or medications	EHR data extraction (baseline, 12mo, 24mo, 36mo)
*Aim 2*		
Insulin sensitivity (primary outcome)	Homeostatic model assessment of insulin resistance (HOMA-IR)	EHR data extraction (baseline and 6 months)
Past 30-day food insecurity	USDA Food Security Survey Module (FFSM)	Quantitative assessment (baseline and 6 months)
Socioeconomic characteristics	Educational attainment, marital status, household composition and size, employment status, income, ratio of household income to poverty	Quantitative assessment (baseline only)
*Nutritional pathway*		
Use of food resources	Seeking a community-based food resource (e.g., food bank or pantry), applying for or receiving Supplemental Nutrition Assistance Program (SNAP), Special Supplemental Nutrition Program for Women, Infants, and Children (WIC), and Temporary Assistance for Needy Families (TANF) benefits	Quantitative assessment (baseline and 6 months)
Food expenditures	Out-of-pocket monthly food expenditures [55]	Quantitative assessment (baseline and 6 months)
Diet quality	Daily fruit and vegetable intake [56]	Quantitative assessment (baseline and 6 months)
Nutrition knowledge	Food literacy [57]	Quantitative assessment (baseline and 6 months)
*Mental health pathway*		
Mental health challenges	Anxiety/stress, depression, substance use [2];\, social support [58]	Quantitative assessment (baseline and 6 months)
Stigma	Internalized stigma related to food insecurity, sexual or gender identity, and/or HIV	Quantitative assessment (baseline and 6 months)
Self-efficacy	Self-efficacy to address food insecurity, manage cardiometabolic comorbidities, and/or engage in HIV care	Quantitative assessment (baseline and 6 months)
*Behavioral pathway*		
Diabetes care	Diabetes self-care activities [59, 60], diabetes self-efficacy [59, 61]	Quantitative assessment (baseline and 6 months)
HIV care engagement	Missed appointments, viral load, medication adherence	EHR data extraction & quantitative assessment (baseline and 6 months)

Data for this aim will be extracted from the EHR, as summarized in Table 1. The primary outcome for Aim 1 is prediabetes or T2DM. A participant will be considered to have a clinical diagnosis of prediabetes if they have a hemoglobin A1C level ≥ 5.7% and < 6.5% measured at the time of the clinic visit. A participant will be determined to have T2DM if they have any one of the following: an A1C ≥ 6.5%, a prior history of T2DM based on ICD-10 codes in the EHR, and/or currently taking a diabetic medication (including glucagon-like peptide 1 receptor agonist, sodium-glucose cotransporter 2 inhibitor, dipeptidyl peptidase 4 inhibitor, sulfonylurea, incretin mimetic, or insulin).

##### Power and sample size for Aim 1

We anticipate accruing 1800 patients (~ 90% of the clinic population) to complete the baseline assessment. These participants will be followed for up to 3 years with an expected loss to follow-up of 15%. Rates of food insecurity in the overall population in our region are 15–20%. In our previous weCare study, about 27% of participants needed support services for food in the past 6 months; in addition, 57% of the clinic population is below 100% of the federal poverty level. Therefore, we assume that 30% of this sample will be food insecure (*n*=540). Based on available data of PWH [55], we expect the baseline prevalence of prediabetes/T2DM in the food-secure group to be 25% at baseline. Under these assumptions, we will have 80% power to detect a difference between a baseline prevalence of prediabetes/T2DM of 25% among non-food-insecure participants and a prevalence of 31.6% (odds ratio=1.38) among food-insecure participants using a two group *χ*^2^ test with a 5% two-sided significance level. Assuming that 75% of the sample will not have prediabetes/T2DM at baseline, the sample size for estimating incidence is *n* = 1350. Based on a study by Nansseu et al. of PWH, we assume the incidence of prediabetes is 125 per 1000 person-years and the incidence of T2DM is 13.7 per 1000 person-years [56]. We then have 80% power to detect a prediabetes incidence rate ratio of 1.7 between those with and without food insecurity and a T2DM incidence rate ratio of 3.7, using a log-rank test for two survival curves with a 5% two-sided significance level.

##### Analysis plan for Aim 1

First, we will compare characteristics of food-secure and food-insecure participants using *t*-tests for continuous covariates and *χ*^2^ tests for discrete covariates. Next, we will estimate the prevalence of prediabetes and T2DM in each group. To compare the unadjusted prevalence of prediabetes and T2DM at baseline for food-secure and food-insecure participants, we will conduct a two-group *χ*^2^ test for two proportions with a 5% two-sided significance level. We will use logistic regression modeling to estimate the association between food insecurity and prevalence of prediabetes and T2DM adjusting for potential confounders such as time since HIV diagnosis, age, gender, and lack of housing. For participants in the longitudinal cohort without prediabetes or T2DM at baseline, we will use survival models for interval-censored data to compare incidence rates between food-secure and food-insecure participants. We will use survival methods for interval-censored data because the exact date of progression to prediabetes or T2DM is unknown but occurs between two assessment dates. In addition, this technique does not need to account for loss to follow-up since it uses models that make use of all available data for those lost to follow-up. We will estimate and compare incidence rates for food-secure and food-insecure participants using the non-parametric survival analysis procedure in SAS PROC ICLIFETEST.

The two groups will be compared with a weighted generalized log-rank test. Next, we will fit proportional hazards regression models for interval-censored data using PROC ICPHREG that will allow us to adjust for potential confounders and include time-varying covariates. Participants will be included in the analysis up until the end of their follow-up (the last time they have an in-person visit) or the first visit in which they are diagnosed with prediabetes or T2DM. For individuals diagnosed with prediabetes at baseline or during the follow-up, we will estimate the incidence of transitioning to diabetes using similar survival models. We will explore the prevalence and incidence of other secondary cardiometabolic comorbidities using similar analyses. We will also use linear mixed effects regression models to estimate trends in A1C and other continuous indicators of cardiometabolic comorbidities (secondary outcomes) over time accounting for the correlation of repeated measures using a random intercept model. We will test for difference in trends (slopes) between food-secure and insecure individuals using *F*-tests. These models will be fit using SAS PROC MIXED.

#### Aim 2: Implement and test weCare/Secure to determine the impact of the intervention on insulin sensitivity among food-insecure PWH with prediabetes or T2DM using an RCT

##### Participants

The participants in Aim 2 will be a subset of Aim 1 participants who enrolled in year 1. From potentially eligible participants identified by EHR data extracted in Aim 1, we will randomly select participants to invite to enroll in Aim 2, starting in year 2. To be eligible to participate in Aim 2, a participant must (a) be a patient of the Wake Forest Infectious Diseases Specialty Clinic, (b) be living with HIV, (c) be receiving antiretroviral therapy (ART) for ≥ 3 months, (d) be ≥18 years of age, (e) screen as food insecure, (f) have prediabetes or T2DM, and (g) provide informed consent. Access to a smartphone will not be an inclusion criterion because participants may communicate with the peer navigator via mHealth platforms accessible on a computer (e.g., Facebook messenger) at their home or in community spaces such as the public library. PWH will be excluded if they are unable to speak English or Spanish, are currently pregnant, are receiving chronic treatment with systemic corticosteroids (similar exclusion as the Look AHEAD Trial, which evaluated the effects of an intervention designed to promote weigh loss among overweight and obese individuals with T2DM [57]), have type 1 diabetes, and/or have cognitive impairment that would prevent participation.

If participants are eligible and consent to participate, they will then complete Aim 2 baseline data collection as outlined in Table 1. After these data are collected, they will be randomly assigned into weCare/Secure (*n*=100) or usual care (*n*=100). Those randomized to the weCare/Secure intervention will receive the intervention for 6 months. For random selection and assignment, our statistician will use block randomization with a randomly selected block size. The research coordinator will enroll, consent, and assign participants to groups. We will continue this process until we reach our sample size of 200. Participants may discontinue participation in the intervention at any time. Given the behavioral nature of the intervention, there will be no other special criteria for discontinuing or modifying allocated interventions. Aim 2 participants will be compensated $40 at baseline and $60 at 6-month follow-up.

##### Intervention overview

In this intervention, the peer navigator will use the mHealth platforms preferred by each participant (i.e., Facebook, Instagram, WhatsApp, texting, and/or GPS-based mobile apps) to communicate one-on-one with each participant in English or Spanish during the 6-month intervention. The peer navigator’s approach is personalized to the needs and priorities of each participant. Table 2 provides an abbreviated summary of peer navigator roles and examples of each role operationalized. The potential for immediate, efficient, and bidirectional in-real-time communication between a peer navigator and each participant is critical to overcome barriers associated with taking action, initiating and maintaining use of food-related resources, and obtaining improved health outcomes, related to both HIV and cardiometabolic disease. Refined messages will be organized by needs (e.g., seeking a food resource such as a pantry; applying for SNAP benefits; overcoming stigma related to food insecurity, sexual or gender identity, and/or HIV; or problem-solving challenges to preparing healthy foods) and by theoretical construct.

**Table 2:** Roles of the peer navigator

Roles	Examples
Check-in	Checks in with the participant periodically to build rapport and trust, provide social support, and maintain bidirectional communication.
Provide information and referrals	Provides information about resources for food. If the referral is to a social service or benefit, the peer navigator will provide information about whether an appointment is needed and if so how to make one.
De-mystify accessing available community-based resources	Explains what to expect when accessing resources, including where to go, transportation options, sign-in processes or the presence of security guards, and whom to ask for.
Explain how to navigate resources and benefits	Explains how to apply for benefits and what materials are needed when applying for SNAP/WIC/TANF benefits.
Send announcements and reminders	Notifies participants of time-sensitive opportunities to access food resources (e.g., a mobile food pantry visiting a specific neighborhood).
Troubleshoot and problem solve	Identifies options for overcoming barriers (e.g., transportation).
Support healthy eating	Provides guidance on shopping for healthy foods on a limited budget and preparing foods according to recommendations for reducing cardiometabolic disease risks (e.g., cooking methods to preserve the nutrient content of vegetables and seasoning foods without high levels of sodium).
Support provider communication	Talks through ways for the participant to share concerns with providers about food insecurity, HIV care, and cardiometabolic disease, including challenges to dietary changes or other cardiometabolic disease management strategies.

##### Usual care

Currently, the standard of care in the Wake Forest Infectious Diseases Specialty Clinic for PWH identified as food insecure is to have them meet with a clinic social worker and review options for food access, such as local food pantries. A resource list is also provided to the PWH. As indicated, referrals for social services may be made. There is no peer navigation within usual care. All participants, those in the weCare/Secure arm and in the usual care arm, will receive usual care. As such, implementing the intervention does not require alteration to usual care pathways (including medication use) and these will continue for both trial arms.

##### Monitoring adherence

The peer navigator will meet with each participant in person at the beginning of their participation in the intervention (after enrollment) to build rapport and lay a foundation of trust that builds during intervention implementation to improve adherence to the intervention. Growing evidence suggests that in-real-time, personalized, and bidirectional social support and problem solving provided by a peer navigator using mHealth may be more successful in changing complex behaviors and behaviors among populations with greater barriers to change (e.g., populations with stigmatized characteristics and/or needs) than a “bot,” a software application structured to provide social media-based messages automatically [46]. We will also conduct process evaluation to document intervention delivery. The peer navigator will maintain a log of their interactions with weCare/Secure participants to capture the mode of intervention delivery, dose, and messaging for each participant (e.g., which mHealth platforms were used, who initiated contact [the participant or peer navigator], the dates and times that messages were sent, number of messages exchanged, and the types of messages utilized to communicate with each participant).

##### Data collection

The quantitative assessment will collect demographic, cognitive, behavioral, psychosocial, and socioeconomic data at baseline and post-intervention follow-up using REDCap (Research Electronic Data Capture), a secure web application for building and managing online surveys and databases maintained by Wake Forest University School of Medicine. Selection of other constructs are guided by the underlying theories and literature describing factors influencing food insecurity and cardiometabolic comorbidities (as highlighted in Fig. 1).

Table 1 provides measurement priorities identified while preparing the application for this study. The assessment, including final selection of measures, will be created using an iterative process by the research team in collaboration with a project-specific community steering committee comprised of community members (including PWH) and representatives from community organizations that provide services related to HIV and to food security. The assessment will be translated into Spanish by a native Spanish speaker trained in professional translation. Participants will complete the quantitative assessment on a tablet while attending baseline and post-intervention (6 months post-baseline) study visits at the clinic.

Our primary outcome for Aim 2 is the difference in mean difference homeostatic model assessment of insulin resistance (HOMA-IR) score between intervention and control arm participants at 6 months, controlling for baseline HOMA-IR is capable of measuring small changes in insulin sensitivity, especially among PWH and persons with prediabetes [58, 59]. At each study visit, fasting glucose and fasting insulin levels will be measured to calculate participants’ HOMA-IR score by venous blood sample. The blood sample will not be stored for future use.

For our secondary outcomes, we will assess for change in food security and change in the pathways (nutrition, mental health, and behavioral; Fig. 2) proposed to influence food insecurity and cardiometabolic comorbidities from baseline to 6-month follow-up (Table 1). To measure the change in food security over a 6-month period, we will use the 18-item USDA Food Security Survey Module (FSSM) 30-day questionnaire, in addition to the 2-item Hunger Vital Sign assessment. The 30-day questionnaire assesses food insecurity over the prior month and successfully assesses changes in food security over time [53, 54].

##### Blinding

As this is an open-label study and it is impossible to blind participants and the peer navigator. Only the data analysts will be blinded (we are using electronic data and an objective measurement -HOMA-IR score) so unblinding will not occur.

##### Provision of ancillary and post-trial care

Participants in the intervention have no greater than minimal risk. There is no anticipated harm or compensation for harm. Any incidental clinical finding will be reported to the participant, if appropriate, and their clinic provider. Participants will maintain regular clinical care post-trial.

##### Process data collection

We will also conduct process evaluation to document intervention delivery. The peer navigator will maintain a log in REDCap of their interactions with weCare/Secure participants to capture the mode of intervention delivery, dose, and messaging for each participant (e.g., which mHealth platforms were used, who initiated contact [the participant or peer navigator], the dates and times that messages were sent, number of messages exchanged, and the types of messages utilized to communicate with each participant).

##### Power and sample size for Aim 2

Assuming 100 participants are randomized to the intervention arm and 100 to the usual care arm, we will have 80% power to detect a difference in the mean change in HOMA-IR between arms of 0.54, assuming a standard deviation of 1.5 (based on unpublished data from our clinic) and a correlation of 0.6 between baseline and follow-up assessments.

##### Analysis plan for Aim 2

We will use a longitudinal RCT with 2 arms (intervention and usual care) to evaluate the impact of the intervention. Data will be analyzed as an intent-to-treat study [60]; with electronic health data, we do not expect much missing data (at any of the time points) so we do not plan to use data imputation techniques. First, we will compare the characteristics of participants assigned to the intervention and usual care arms using *t-*tests for continuous covariates and *χ*^2^ tests for discrete covariates. Our primary data analysis will compare mean HOMA-IR levels for the intervention and usual care arm participants at the 6-month follow-up assessment adjusting for baseline levels. This follow-up adjust baseline approach has the advantage of being unaffected by baseline differences. If baseline rates, by chance, were different in the intervention arm, the intervention effect would be overestimated by looking at change scores and underestimated by a follow-up score analysis. The ANCOVA approach gives the same answer whether or not there is baseline imbalance. Additionally, this approach generally has greater statistical power to detect an intervention effect than the other methods. Statistical analyses will be performed using linear models for continuous outcomes in SAS PROC GLM.

#### Aim 3. Advance our understanding of how weCare/Secure can most effectively improve the management and treatment of cardiometabolic comorbidities among food-insecure PWH

##### Participants

The interview participants in Aim 3 will be a subset of Aim 2 participants and recruited from three groups of Aim 2 intervention participants: (a) participants who reduced both food insecurity and increased insulin sensitivity (*n* = 15); (b) participants who either reduced food insecurity or improved insulin sensitivity (but not both; *n* = 15); and (c) participants who did not reduce food insecurity or improve insulin sensitivity (*n* = 15). Participants will be randomly selected by group. The sample size of 45 interview participants should be adequate to reach thematic saturation across these groups, but we will have flexibility to expand this sample if warranted. We will purposely recruit a diverse sample by age, time since HIV diagnosis, gender, race/ethnicity, sexual orientation, and home address (urban or rural). Aim 3 participants will be compensated $50 for their time.

##### Data collection

After the 6-month follow-up, we will conduct interviews to advance our understanding of how the intervention can improve management of cardiometabolic comorbidities with PWH. A semi-structured interview guide will be designed to assess the strengths and weaknesses of the intervention, understand participant experiences, contextualize the intervention’s impact, identify lessons learned, guide any intervention revisions, and inform future research directions. It also will focus on how the intervention affected the three pathways outlined in Fig. 2, and how interventions to address food insecurity in clinics caring for PWH can most effectively be tailored. Using the same process as above, the interview guide will be translated into Spanish.

##### Analysis plan for Aim 3

The interviews will be digitally recorded and professionally transcribed (and translated if in Spanish), with identifying data removed. Analyses will be guided by the constant comparison method, informed by grounded theory methodologies [61, 62]. This approach is well suited for systematically uncovering participant experiences and comparing them within and across groups (e.g., age, time since HIV diagnosis, gender, race/ethnicity, sexual orientation, and urban/rural). Analytic steps will include the following: after each interview, interviewers will document field notes including emerging topic areas for subsequent exploration; transcripts and field notes will be entered into Atlas.ti qualitative data management software (Berlin, Germany) for coding and analysis; we will develop a preliminary codebook to include pre-determined deductive codes related to the 3 domains (nutrition, mental health, and behavioral) and inductive codes that emerge from the data; coding differences will be discussed and negotiated in team meetings until consensus is reached; similarities and differences across transcripts will be examined and themes developed accordingly; and we will “member check” themes with the steering committee to refine and establish the validity of the results. From this rich dataset, we will develop a conceptual understanding of how interventions to address food insecurity can most effectively improve cardiometabolic comorbidities among PWH.

## Monitoring

### Steering committee

The study will be conducted by the study investigators who will be responsible for all aspects of the local organization including identifying potential participants and obtaining consent. The trial steering committee is comprised of clinic staff, and racially/ethnically and gender-diverse PWH, including individuals at risk for food insecurity and those living in rural areas. Given the diversity of the clinic, it is critical to ensure that we obtain a broad range of perspectives throughout this process. The trial steering committee will meet monthly for the first year of this study and quarterly thereafter to provide guidance on mHealth messages, measurement, recruitment and retention, and interpretation of findings.

### Data Safety and Monitoring Board (DSMB)

The DSMB will be comprised of five external researchers and a representative from a local food pantry. The DSMB will provide another mechanism to ensure the safe conduct of the study and provide additional scientific guidance. This DSMB will meet at least once annually, and more often as needed either in person or virtually to audit trial conduct.

On an ongoing basis, adverse events or complaints about the study will be reported to the study team and the DSMB. Given the behavioral nature of the intervention, serious adverse events are not anticipated; any serious adverse events and/or unanticipated problems involving risks will be reported to the DSMB, institutional officials, and sponsor as required by the protocol and federal regulation. The Principal Investigators will convene the DSMB if any member regards any adverse event as serious enough to discuss and any patterns of complaints or concerns expressed by participants. Follow-up options for adverse events include the following: (a) DSMB members will determine that the event(s) are serious enough to recommend immediate discontinuation of the study. In this case, all ongoing study participants will be notified as soon as possible of the study termination. Such notification will be initiated within 24 h of the DSMB decision; (b) DSMB members will determine that the events are serious enough to notify all participants enrolled and any future eligible participants about the nature of the event and expected consequences for remaining enrolled or initiating enrollment; and (c) DSMB members will determine that events are not serious enough to notify participants and potential enrollees, and the study enrollment and follow-up will proceed as outlined in the study protocol.

## Trial status

Aim 1 (longitudinal cohort) recruitment began in May 2022 and is ongoing. Aim 2 (intervention) recruitment is planned for spring 2023 and is expected to be completed in spring 2024. Aim 3 (process evaluation) data collection will occur after sufficient completion of the 6-month assessment in Aim 2. Final results are anticipated in fall 2025.

## Discussion

The outlined research seeks to advance our understanding of how food insecurity impacts the development of cardiometabolic comorbidities among PWH and how interventions for food insecurity may alleviate relevant comorbidities. Given the growing interest among health systems in addressing food insecurity as a routine part of clinical practice, if the intervention is found to be efficacious, it could be broadly disseminated across HIV clinical care settings.

## Data Availability

De-identified data and associated documentation will be available from the Principal Investigators under a data-sharing agreement with users that provides for a commitment to using the data for research purposes, a commitment to securing the data using appropriate computer technology, and a commitment to destroying or returning the data after analyses are completed. Informed consent forms are available from the corresponding author by request.

## Abbreviations

ART: Antiretroviral therapy
EHR: Electronic health record
HOMA-IR: Homeostatic model assessment of insulin resistance
IRB: Institutional Review Board
PWH: People living with HIV
SNAP: Supplemental Nutrition Assistance Program
SDH: Social determinants of health
TANF: Temporary Assistance for Needy Families
T2DM: Type 2 diabetes mellitus
WIC: Special Supplemental Nutrition Program for Women, Infants, and Children
